# Fully Automated Segmentation Models of Supratentorial Meningiomas Assisted by Inclusion of Normal Brain Images

**DOI:** 10.3390/jimaging8120327

**Published:** 2022-12-15

**Authors:** Kihwan Hwang, Juntae Park, Young-Jae Kwon, Se Jin Cho, Byung Se Choi, Jiwon Kim, Eunchong Kim, Jongha Jang, Kwang-Sung Ahn, Sangsoo Kim, Chae-Yong Kim

**Affiliations:** 1 Department of Neurosurgery, Seoul National University Bundang Hospital, Seoul National University College of Medicine, Seongnam-si 13620, Gyeonggi-do, Republic of Korea; 2 Department of Bioinformatics, Soongsil University, Seoul 06978, Republic of Korea; 3 Seoul National University College of Medicine, Seoul 03080, Republic of Korea; 4 Department of Radiology, Seoul National University Bundang Hospital, Seoul National University College of Medicine, Seongnam-si 13620, Gyeonggi-do, Republic of Korea; 5 Department of Functional Genome Institute, PDXen Co., Daejeon 34027, Republic of Korea; 6 Cancer Research Institute, Seoul National University College of Medicine, Seoul 03080, Republic of Korea

**Keywords:** meningioma, magnetic resonance imaging, deep learning, U-net

## Abstract

To train an automatic brain tumor segmentation model, a large amount of data is required. In this paper, we proposed a strategy to overcome the limited amount of clinically collected magnetic resonance image (MRI) data regarding meningiomas by pre-training a model using a larger public dataset of MRIs of gliomas and augmenting our meningioma training set with normal brain MRIs. Pre-operative MRIs of 91 meningioma patients (171 MRIs) and 10 non-meningioma patients (normal brains) were collected between 2016 and 2019. Three-dimensional (3D) U-Net was used as the base architecture. The model was pre-trained with BraTS 2019 data, then fine-tuned with our datasets consisting of 154 meningioma MRIs and 10 normal brain MRIs. To increase the utility of the normal brain MRIs, a novel balanced Dice loss (BDL) function was used instead of the conventional soft Dice loss function. The model performance was evaluated using the Dice scores across the remaining 17 meningioma MRIs. The segmentation performance of the model was sequentially improved via the pre-training and inclusion of normal brain images. The Dice scores improved from 0.72 to 0.76 when the model was pre-trained. The inclusion of normal brain MRIs to fine-tune the model improved the Dice score; it increased to 0.79. When employing BDL as the loss function, the Dice score reached 0.84. The proposed learning strategy for U-net showed potential for use in segmenting meningioma lesions.

## 1. Introduction

Meningiomas are tumors in the meninges that cover the brain and spinal cord. As many of them are asymptomatic, they are often accidentally detected during magnetic resonance imaging (MRI) examinations, for example, during routine medical check-ups. Patients who experience incidental meningioma discovery undergo routine MRI scans to monitor the tumor’s growth. Two-dimensional measurements of tumors can potentially underestimate the risk of tumor growth. In comparison, volumetric measurements can enable the tumor growth to be monitored with high accuracy.

However, the manual measurement of tumor volume is a laborious task, making treatment planning challenging. There is variability in measurement due to (1) varying expertise levels between radiologists and (2) inherent human errors. Hence, the automation of tumor segmentation is imperative for tumor monitoring.

There has been substantial progress in the field of 3D medical image segmentation based on deep learning, especially with the advent of U-Net [1], because U-Net is able to learn feature maps from many slices. Since then, there have been breakthrough studies stemming from U-Net [2,3,4]. Due to the specificities of data representation from image to sentence, we are very aware that certain deep learning structures perform better than others [5]. The attention mechanism is very popular in the field of Natural Language Processing (NLP), because it has allowed us to enrich the input data features and guide the neural architecture to enable more relevant elements to be found [6].

There have been attempts to incorporate the attention module to U-Net for use in medical image segmentation [2,3,7,8]. Yeung et al. introduced novel dual attention-gated U-Net architecture, called Focus U-Net, for use in polyp segmentation in colonoscopy images [2].

To train any model, a large dataset, good model, and a well-defined loss function and optimizer are needed [9]. Firstly, it is challenging to collect a good amount of medical imaging dataset. In machine learning communities, transfer learning from another domain is a conventional strategy. Transfer learning is widely used to overcome this limitation.

The glioma dataset from the purpose of the Brain Tumor Segmentation (BraTS) benchmark [10,11,12] has been used to evaluate various state-of-the-art segmentation methods. While glioma segmentation methods are being actively studied using the BraTS benchmark [13,14,15,16], relatively few methods have been reported for use in meningioma segmentation, especially from MRI images. 

The utilization of gliomas from the BraTS dataset in order to enable meningioma segmentation is a domain adaptation problem. Effort has been made to overcome such a problem: Ouyang et al. [17] achieved a state-of-the-art performance in 3D CT medical image segmentation when the model was pre-trained with a different modality: 3D MRI.

Recently, Laukamp et al. [18] successfully segmented lesions in meningioma patients using a three-dimensional (3D) neural network (CNN) trained solely with the BraTS benchmark. Later, Laukamp et al. [19] demonstrated an improved meningioma segmentation model which was trained using the same 3D CNN but with meningioma MRI images alone. It was postulated that training models with matched tumor types was superior to borrowing a model developed for a different tumor type. Bouget et al. reported a meningioma segmentation model using a large dataset, which achieved good overall performance, while its performance was compromised when used for small tumors [20].

In numerous brain tumor segmentation studies [18,19], the structures of the lesions are typically classified into the categories of contrast-enhancing tumors, non-contrast-enhancing tumors, necrosis, and edema. However, meningioma lesions are much more clinically diverse, ranging from solid tumors to tumors with necrosis, edema, cysts, calcification, or heterogeneous enhancement. Such diversified lesions are expected to hinder the efficient training of neural networks, as these structures are assumed to be noisy. Hence, previous studies have only focused on defined lesions [18,19]. To reflect the actual diversity of real-world data in the clinic, we used meningioma data containing diverse radiological findings to build an automatic deep-learning-based segmentation model.

Recently, the fine-tuning of U-Net-structured neural networks (TernausNet) pre-trained using large amounts of data such as ImageNet [21] has provided good performances in two-dimensional (2D) medical image segmentation [17]. A model built from a non-medical domain has fared well in this task; however, enhanced model performance can be expected if we train a model using medical images. Inspired by previous studies [16,17,18,19], we attempted to utilize a model that was trained with BraTS glioma images. We chose to use nnU-net, which was proposed by Isensee et al. [22], as the neural network structure. Then, we attempted to extend the definition of soft Dice loss, proposed by Milletari et al. [23], in order to incorporate brain MRI images without lesions; we named this balanced Dice loss (BDL). Finally, we implemented an Adam optimizer to minimize the loss function.

In this paper, we reported ablation studies regarding the training strategies used when only scarce medical datasets were available. An automated meningioma segmentation model was made using a series of steps: transfer learning with BraTS glioblastoma and fine-tuning with meningioma and radiologically clean brain images. We implemented a modified version of soft Dice loss for an nnU-net model [22] to enable the model to learn all of the features from our dataset.

## 2. Materials and Methods

### 2.1. Study Approval

The study complied with the Declaration of Helsinki. The Institutional Review Board of Seoul National University Bundang Hospital, SNUBH, approved the current study (No. B-2006-616-106) and waived the requirement for written informed consent.

### 2.2. Patients

Between 2016 and 2019, MRIs of supratentorial meningiomas were collected through medical check-ups (Tables S1 and S2). Moreover, normal brain MRI scans from 10 subjects were also obtained. All of the MRI scans were performed in a single tertiary hospital. MRI evaluations of the enrolled patients were performed using either a 1.5 T (Megatom Amira; Siemens Healthcare Systems, Erlangen, Germany) or 3.0 T (Ingenia, Achieva; Philips Medical Systems, Best, The Netherlands) system. Each MRI protocol for supratentorial meningioma comprised four sequences: an axial T1-weighted image (T1WI), a three-dimensional axial contrast-enhanced T1WI (3D CE-T1WI), an axial T2-weighted image (T2WI), and axial T2-fluid attenuated inversion recovery (FLAIR) (see S1 File for further information). In the 1.5 T scanner, the axial T1WI was obtained with a repetition time/echo time (TR/TE) of 500/7.3 ms; a flip angle (FA) of 80°; an acquisition matrix of 320 × 235 mm^2^; a slice thickness of 5 mm; and section spacing of 6 mm. The axial T2WI was obtained with a TR/TE of 3480/97 ms; an FA of 160°; an acquisition matrix of 384 × 327 mm^2^; a slice thickness of 5 mm; and section spacing of 6 mm. Axial FLAIR was obtained with a TR/TE of 8000/126 ms; an FA of 150°; an acquisition matrix of 320 × 232 mm^2^; a slice thickness of 5 mm; and section spacing of 6 mm. The sagittal 3D CE-T1WI was obtained with a TR/TE of 600/3.8 ms; an FA of 120°; an acquisition matrix of 256 × 256 mm^2^; a slice thickness of 1 mm; and without section spacing. In the 3 T scanner, the axial T1WI was obtained with a TR/TE of 600/7.3 ms; an FA of 90°; an acquisition matrix of 256 × 256 mm^2^; a slice thickness of 5 mm; and section spacing of 6 mm. The axial T2WI was obtained with a TR/TE of 3000/80 ms; an FA of 90°; an acquisition matrix of 400 × 294 mm^2^; a slice thickness of 5 mm; and section spacing of 6 mm. Axial FLAIR was obtained with a TR/TE of 11,000/125 ms; an FA of 90°; an acquisition matrix of 240 × 240 mm^2^; a slice thickness of 5 mm; and section spacing of 6 mm. The sagittal 3D CE-T1WI was obtained with a TR/TE of 10.46/6.91 ms; an FA of 8°; an acquisition matrix of 240 × 240 mm^2^; a slice thickness of 1 mm; and without section spacing. The region of interest for meningioma was generated by two radiologists (S.J.C. and B.S.C., with 7 and 20 years of experience in neuroradiology, respectively).

The 171 meningioma MRI datasets consisted of 115 follow-up MRIs for 35 patients and 56 non-follow-up MRIs for 56 patients. Intact brains were obtained from lung cancer patients who had undergone a metastasis check-up, but there was no extra-pulmonary metastasis at all. Hereafter, intact brains are referred to as normal brains.

### 2.3. Pre-Processing of MRI

Well-known tools, such as the FMRIB Software Library (FSL) [24] and Advanced Normalization Tools (ANTs) [25], were used to pre-process the brain MRIs. As the segmented tumors usually resided on the border of the brain, we confirmed that the brain extraction tool had not removed any lesions. The reorientation process of changing the direction to match the direction of the Montreal Neurological Institute and Hospital (MNI) standard image was performed using FSL. Co-registration, bias field correction, and brain extraction were performed using ANTs to match the coordinate system of the image. All MRIs were normalized and resized to 128 × 128 × 128 (vide infra). The ground-truth masks of meningioma lesions were manually labeled by two radiologists on the pre-processed T1CE images, because T1CE images usually have better resolutions (10×) than T2-W images. Each voxel with the lesion volume was labeled as 1, and the rest were labeled as 0. All of the voxels in the normal brain MRI scans were labeled with 0.

### 2.4. Three-Dimensional Neural Network (3D U-Net)

The U-Net [1] structure is popular for image segmentation. It has been successfully extended to handle 3D images, such as the BraTS 2015 dataset by Kayalibay et al. [26]. Isensee et al. [22] further developed a 3D U-Net to more efficiently handle large images. For example, this network architecture uses approximately twice as many filters as those in the architecture designed by Kayalibay et al. [27] by optimizing the number of feature maps. It also uses localization paths to reduce memory consumption and instance normalization [27], because batch normalization can become unstable with a small batch size. Furthermore, the activation function across the network uses a leaky rectified linear unit (ReLU) [28].

We adopted the 3D U-Net proposed by Isensee et al. [22] as the neural network structure. We used the relevant source codes implemented in Keras [29] and Tensorflow [30] by Ellis and Aizenberg, which are available on Github [31]. Training for this network was carried out on a single 8G graphics processing unit (GPU) on an NVIDIA GeForce RTX 2080 graphics card environment. The maximum input image size was 128 × 128 × 128 pixels.

### 2.5. Loss Function

The loss function for training used a metric based on the Sørensen–Dice coefficient (Dice similarity coefficient (DSC)) proposed by Pelicer [32]. The DSC is an overlap metric often used to evaluate the quality of segmentation maps. This is defined in Equation (1):

(1) $$DSC=\frac{2\Sigma P\cdot T\;}{\Sigma P+\Sigma T}$$

where *P* is the output of the network and *T* is the ground truth. Proposed by Milletari et al. [33], soft Dice loss using the *DSC* has been used as a loss metric in numerous studies. A small constant, *ϵ*, was added to the numerator and denominator to prevent the denominator from becoming zero and smoothing the function; that subtracted from 1 was used as the loss function, as shown in Equation (2).

(2) $$L_{Dice}=1-\frac{2\Sigma P\cdot T+\epsilon }{\Sigma P+\Sigma T+\epsilon }$$

However, a soft Dice loss with a typically very small *ϵ* becomes close to 1 for data in which the sum of the weights is forced to be 0 (∑*T* = 0), and it is not very sensitive to changes in the network output (∑*P*). This occurred in the normal brain images, in which all of the pixels were labeled with 0. To emphasize the contribution from the normal data, the weight, *β*, was multiplied when ∑*T* = 0. The balanced Dice loss (BDL) (*L_BD_*) is defined as:

(3) $$\;L_{BD}=1-\left(\alpha L_{Dice}\left(T,P\right)+\beta \left(1-\alpha \right)L_{Dice}\left(T,P\right)\right)$$

where α represents max (*T*). As the ground truth is a binary label consisting of 0 and 1, α = 1 in the case of a tumor, and *α* = 0 in the case of normal data. Hyperparameter *β* was optimized during training. In this study, *L_Dice_* was used as the loss function to learn a dataset consisting of brain tumor MRIs alone, whereas *L_BD_* was used to learn a dataset containing normal data. The final loss was the sum of losses in all of the images.

### 2.6. Model Training and Selection

The 3D U-Net model was trained by minimizing the Dice-based loss function described above. For minimization, an Adam optimizer [34] was used with an initial learning rate of 10^−4^, and the learning rate was reduced by half every 30 epochs. The segmentation performance of the model at each epoch was evaluated using the Dice score defined by Equation (1). To avoid overfitting, the training was quantitatively evaluated via five-fold cross-validation (CV), in which the scores of each fold were averaged. The epoch with the minimal averaged Dice-based loss was used to obtain the final model, which was then trained with all of the training data without CV.

## 3. Results

Our meningioma dataset included the MRI scans of follow-up patients. To prevent the inclusion of the same patient’s MRIs in both the training and test sets, the test set (17 MRIs) was randomly extracted from non-follow-up cases. The average tumor volume of the test set meningioma was 30.31 cm^3^ (minimum: 0.24 cm^3^, maximum: 139.87 cm^3^) according to the experts’ manual segmentation (Figure S1). We employed the training strategies by varying the data used for (1) pre-training and (2) fine-tuning. A five-fold CV was used for hyperparameter selection. The test set was fixed for all strategies, and we reported the performance scores based on this test set.

As shown in Table 1, the 3D U-Net, trained with a meningioma dataset, achieved a higher Dice score of 0.72 (sd: 0.28) than the Dice score of 0.60 (sd: 0.32) which was achieved with the BraTS 2019 dataset. As reported by Laukamp et al. [18,19], the performance increased when the neural network was trained with the disease of interest, that is, meningioma. This implies that transfer learning [35] from one disease to another requires fine-tuning with the latter. Indeed, pre-training with BraTS 2019 followed by fine-tuning with a meningioma dataset increased the Dice score to 0.76 (sd: 0.23). It appears that pre-training not only stabilizes the training process but also contributes to learning parts that are not learned in the existing dataset.

**Figure 1:**
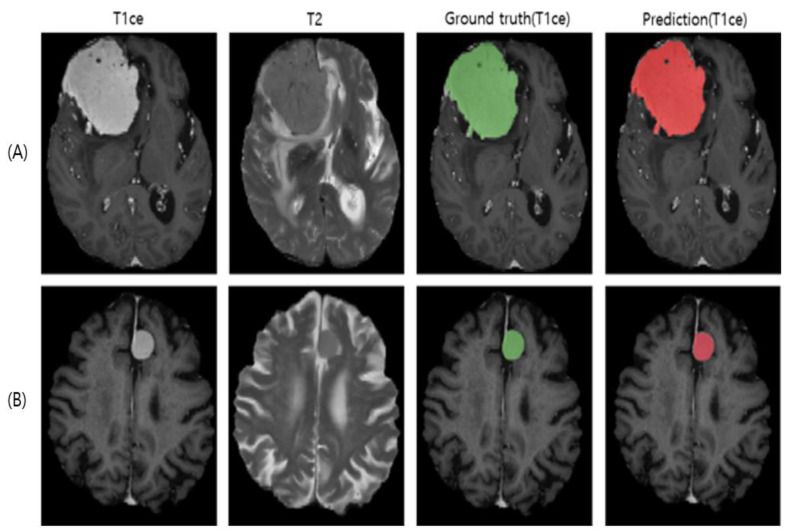
Representative meningioma segmentation models learned using transfer learning, normal brain MRIs, and balanced Dice loss (BDL). Two patients (**A**,**B**) were from the test set. Note that the ground truth was generated based on T1ce.

We also evaluated the use of normal brain data during training. This increased the sample size from 74 to 84. Transfer learning and the use of normal brain data increased the Dice score to 0.79 (sd: 0.23). However, the soft Dice loss function did not properly account for the contribution from normal data, where the losses remained close to 1. Our BDL could give more weight to normal data by adjusting hyperparameter *β*. Using a five-fold CV, the β was optimized to 100. As a result, we achieved a Dice score of 0.84 (sd: 0.15) with the test dataset. The average segmentation performance across all folds was 0.85 (sd. 0.04) (Dice scores of each fold: 0.88, 0.82, 0.86, 0.92, and 0.80), confirming the stability of the model. Although our test set was limited, its performance was very similar to the stable performance of the larger training set. Hence, it seems that there was no overfitting issue. Two representative examples of the segmentation results of the final model (transfer learning + normal + BDL) are shown in Figure 1. The Dice scores for these two subjects were 0.96 (Figure 1A) and 0.93 (Figure 1B).

## 4. Discussion

In this study, deep learning was used for the fully automated segmentation of supratentorial meningiomas. To overcome the fact that there was a relatively small amount of meningioma image data, transfer learning with a large number of publicly available BraTS glioma images was used to produce the initial model for meningioma segmentation. Then, MRIs consisting of both meningiomas and normal brains were included in the fine-tuning of the final model.

Typical meningiomas appear as dural-based masses that are isointense to gray matter on both T1- and T2-weighted images. To the best of our knowledge, previous studies have only focused on well-defined meningioma MRI samples for the development and evaluation of such models [18,19], while there are large variations in real-world imaging appearance [35].

To reflect such issues, we gathered sets of meningioma MRI images with diverse characteristics, including cysts, calcifications, necrosis, and heterogeneously enhancing lesions. We focused our model on learning the features of supratentorial meningioma, as infratentorial meningioma is relatively rare and intermingled with complicated neurovascular structures. As this study is the first trial to assess the utility of automatic segmentation for meningioma, we simplified our MRI dataset.

Bouget et al. investigated automated meningioma segmentation using only one imaging modality (T1) with a lightweight model [20]. However, it had a severe drawback: a dip in the Dice score (~0.5, at best) when the meningioma lesion was smaller than 1 cm^3^. Small tumors should not be ignored, because tumor growth rates are unpredictable. To assess the consistency in performance across the tumor size, we categorized the tumor sizes into three levels from smallest to largest, creating Dice score boxplots (Figure S2). Our model showed a modest decrease in performance for small-sized tumors (~0.7 for Category A tumors (<0.4 cm^3^)).

**Figure 2:**
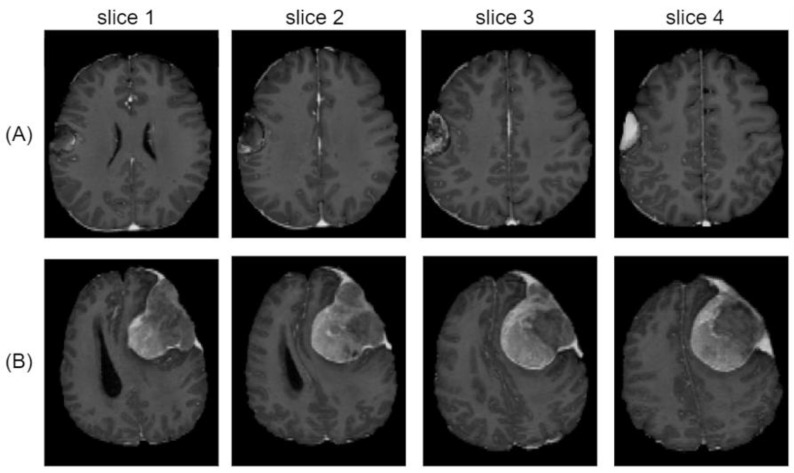
Meningioma lesion from two representative patients in the test set. (**A**,**B**) are brain slices showing heterogeneous enhancement inside the tumor (15 mm spaced slices).

Although our model showed good performance in clinically diverse lesions, the frequencies of such appearances were limited in our dataset. Hence, the performance of the test samples fluctuated to some extent. It was especially poor in cases of meningioma with heterogeneous enhancement. As shown in Figure 2A, heterogeneous enhancement due to necrosis was observed inside the tumor, and the predictive performance of the model for this subject had a Dice score of 0.34. Heterogeneous enhancement was also observed in Figure 2B, and the predictive performance of the model for this brain was a Dice score of 0.84. To overcome this issue, brain images including heterogeneous enhancement lesions should be collected and used to train models. If the model matures enough to handle the primary task—meningioma segmentation—then we believe that the model can be further improved, which would also help explain various features of meningioma [36]. 

While most other studies have mainly focused on a model’s architecture to improve its performance, our proposed strategy involved transfer learning and the inclusion of normal brain MRIs. To effectively utilize normal MRIs, we successfully developed a new loss function, BDL. Notably, in our study, better performance was achieved after the inclusion of normal cases in the training set.

There are some limitations to our study. Our study only included images obtained using an MRI dataset from a single institution. Previous studies have attempted to acknowledge inter-hospital or inter-protocol variability by introducing images from multiple institutions or from multiple scanners. As our model only used data sources from a single institution, it might have lost generalization and thus requires data collection involving multiple institutions or scanners.

Realistic data curation was performed to address how deep learning can be used to expedite the meningioma segmentation process. We used nnU-Net along with the most renowned optimizers (i.e., Adam and/or LeakyRelu optimizers). However, we could use different sets of optimizers and loss function to determine the robustness of the model. There are many recent studies that suggest the possibility of improvements, such as the AdamP by Heo B. et al. [37]. Performance gain is the expected result, because several studies have explained the link between optimizer and model performance [38]. Additionally, in the future, we could determine the loss function, which is especially built for tasks such as meningioma segmentation. Class imbalance, where the lesion volume is much smaller than the whole brain volume, affects the model performance. However, we could try to use Unified Focal loss, which is able to handle class imbalance [39].

## 5. Conclusions

In our study, we proposed a learning strategy for the fully automated segmentation of meningioma containing clinically diverse types of lesions; we also proposed that the inclusion of normal brain datasets through a deep learning algorithm could improve segmentation performance. This study will serve as the basis for tumor detection or automated tumor volume measurement in clinical settings in future studies. In addition, the findings of the present study and the data reported could serve as a foundation for evaluating the growth patterns of meningiomas.

## Figures and Tables

**Table 1:** Model inference performance. All Dice scores are averaged against the test set (17 MRIs). [A] Averaged Dice: 0.60 (sd: 0.32), range: [0.00, 0.97]. [B] Averaged Dice: 0.72 (sd: 0.28), range: [0.09–0.98]. [C] Averaged Dice: 0.76 (sd: 0.23), range: [0.25–0.95]; [D] Averaged Dice: 0.79 (sd: 0.26), range: [0.00–0.98]; [E] Averaged Dice: 0.84 (sd: 0.15), range: [0.37–0.97].

Methods	Training Set	Patients	Total MRIs	Averaged Dice (sd)	Recall (sd)	Precision * (sd)
[A] BraTS	BraTS	335	335	0.60 (0.32)	0.64 (0.35)	0.71 (0.37)
[B] Meningioma	Meningioma	74	154	0.72 (0.28)	0.83 (0.29)	0.78 (0.27)
[C] TL	BraTS (pre-training)	335	335	0.76 (0.23)	0.79 (0.29)	0.84 (0.19)
	Meningioma	74	154			
[D] TL + Normal	BraTS (pre-training)	335	335	0.79 (0.26)	0.82 (0.28)	0.81 (0.29)
	Meningioma	74	154			
	Normal	10	10			
[E] TL + Normal + BDL	BraTS (pre-training)	335	335	0.84 (0.15)	0.89 (0.18)	0.84 (0.15)
	Meningioma	74	154			
	Normal	10	10			

* Performances for all training strategies were measured on a common test set (17 MRIs). BDL, balanced Dice loss; TL, transfer learning.

## Data Availability

All brain image files, formatted as nii.gz, are available from the Zenodo database (doi: 10.5271/zenodo.5945963).

## Supplementary Materials

The following supporting information can be downloaded at: https://www.mdpi.com/article/10.3390/jimaging8120327/s1, File S1: Supplementary file detailing MRI acquisition methods; Table S1: Table of enrolled subjects’ characteristics; Table S2: Table of enrolled subjects’ meningioma characteristics; Figure S1: Violin Plots of whole tumor volume; Figure S2: Dice score plots against binned whole tumor size.
