# Supplementary material for: Fully Automated Segmentation Models of Supratentorial Meningiomas Assisted by Inclusion of Normal Brain Images
Source: J Imaging. 2022 Dec 15;8(12):327. doi: 10.3390/jimaging8120327 (PMC9782766; doi:10.3390/jimaging8120327)
Supplement: Supplementary file 1 [file jimaging-08-00327-s001.zip › jimaging-1972887-supplementary.pdf]

File S1. Supplementary file detailing MRI acquisition methods.

MRI evaluations of the enrolled patients were performed either on the 1.5-T (Megatom Amira; Siemens Healthcare Systems, Erlangen, Germany) or a 3.0-T system (Ingenia, Achieva; Philips Medical Systems, Best, the Netherlands). Each MRI protocol for supratentorial meningioma encompasses four sequences: axial and sagittal T1-weighted images (T1WI), three-dimensional axial, sagittal, and coronal contrast-enhanced T1WI (3D CE-T1WI), axial T2-weighted image (T2WI), and axial T2-Fluid Attenuated Inversion recovery (FLAIR). In the 1.5-T scanner, axial T1WI was obtained with a repetition time/echo time (TR/TE), 500/7.3 ms; flip angle (FA), 80°; acquisition matrix, 320 × 235 mm^2; slice thickness of 5 mm, and section spacing of 6 mm. Axial T2WI was obtained with a TR/TE of 3480/97 ms, FA of 160°, acquisition matrix of 384 × 327 mm^2, slice thickness of 5 mm, and section spacing of 6 mm. Axial FLAIR images were obtained with a TR/TE of 8000/126 ms; FA, 150°; acquisition matrix, 320 × 232 mm^2; slice thickness, 5 mm; and section spacing, 6 mm. Sagittal 3D CE-T1WI was obtained with a TR/TE, 600/3.8 ms; FA, 120°; acquisition matrix, 256 × 256 mm^2; slice thickness of 1 mm, without section spacing. In the 3-T scanner, axial T1WI was obtained with a TR/TE, 600/7.3 ms; FA, 90°; acquisition matrix, 256 × 256 mm^2; slice thickness of 5 mm, and section spacing of 6 mm. Axial T2WI was obtained with a TR/TE of 3000/80 ms; FA, 90°; acquisition matrix, 400 × 294 mm^2; slice thickness, 5 mm; and section spacing, 6 mm. Axial FLAIR images were obtained with a TR/TE of 11000/125 ms; FA, 90°; acquisition matrix, 240 × 240 mm^2; slice thickness, 5 mm; and section spacing, 6 mm. Sagittal 3D CE-T1WI was obtained with a TR/TE, 10.46/6.91 ms; FA, 8°; acquisition matrix, 240 × 240 mm^2; slice thickness of 1 mm, without section spacing. The region of interest for meningiomas was generated by two radiologists (S.J.C. and B.S.C., with 7 and 20 years of experience in neuroradiology, respectively).

Table S1. Table of enrolled subjects' characteristics

		Patients (n=91)	Normal (n = 12)	Total (n= 103)
Sex	Female	66	5	71
	Male	25	7	32
Age (years)	Mean	60.1	56.9	58.5
	Range	[19,92]	[32, 80]	[19,92]

Table S2. Table of enrolled subjects' meningioma characteristics

		Numbers	Percentage (%)
Localization	Convexity	48	53
	Parasagittal	10	11
	Falx	10	11
	Sphenoid wing	10	11
	Tentorial	6	7
	Olfactory groove	3	3
	Tuberculum sellae	2	2
	Clinoidal	1	1
	Intraventricular	1	1
	-	-	-
	-	-	-
Attachment to Skull Base	Yes	22	24
	Total	91	100

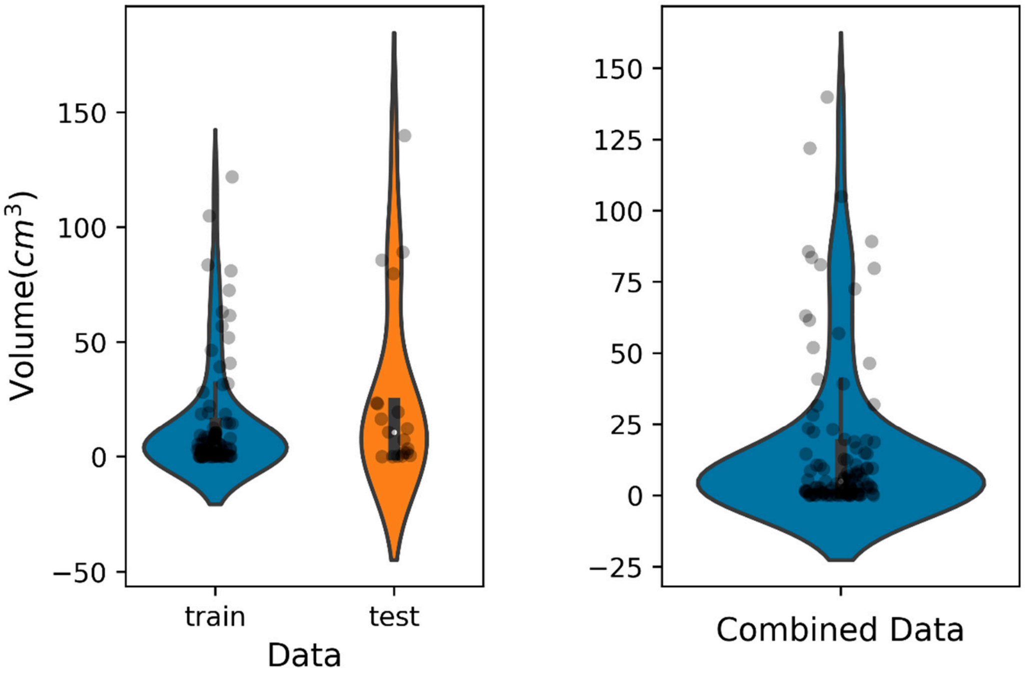

Figure S1. Violin Plots of whole tumor volume. The dataset is grouped as train/test (left panel) and combined (right panel).

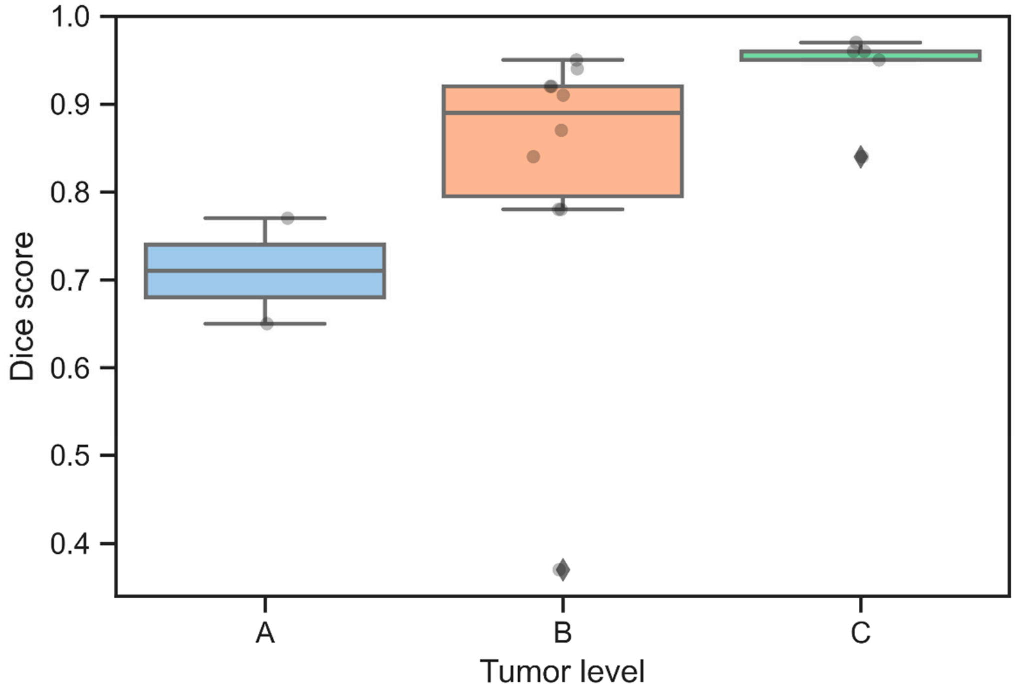

Tumor Level	Size Range (cm^3)
A	0 - 0.4
B	0.4 - 23.355
C	23.355 - 139.87

Figure S2. Dice score plots against binned whole tumour size. Binning of whole tumor size has been done with alphabetically ascending order so that performance (i.e. Dice score) on the test set can be compared across size.
